# A real time action scoring system for movement analysis and feedback in physical therapy using human pose estimation

**DOI:** 10.1038/s41598-025-29062-7

**Published:** 2025-12-29

**Authors:** Rahmat Ullah, Ikram Asghar, Rab Nawaz, Craig Stacey, Saeed Akbar, Peter Bishop

**Affiliations:** 1 School of Computer Science and Electronic Engineering, University of Essex, Colchester, UK; 2 Department of Computing and Games, Teesside University, Middlesbrough, UK; 3 CEMET, University of South Wales, Pontypridd, UK; 4 Department of Physical and Numerical Sciences, Qurtuba University of Science and Information Technology, Peshawar, Pakistan; 5 Augmented Vision Limited, Wales, UK

**Keywords:** Rehabilitation, Computer science

## Abstract

Human Pose Estimation (HPE) has become an essential tool in physical therapy, enabling automated movement analysis and rehabilitation monitoring. However, existing HPE techniques often suffer from limitations such as motion blur, occlusions, inconsistent keypoint visibility, and sensitivity to variations in camera angles and subject positioning. This makes the movement assessment very challenging, particularly in home-based rehabilitation settings where real-time supervision is limited. To address these issues, a novel action-scoring algorithm that integrates angular-based movement analysis with keypoint normalization techniques is presented in this study. Specifically, the proposed method employs Dynamic Time Warping (DTW) and Normalized Cross-Correlation (NCC) for precise movement comparison, alongside a fixed bounding box strategy to improve tracking stability. Additionally, a new repetition counting mechanism based on angular calculations is introduced to ensure accurate assessment of repetitive exercises. The proposed approach primarily aims to minimize the angular noise such as motion blur. Hence, the proposed system can be easily integrated with existing human pose estimation systems. Experimental validation demonstrates that the proposed approach achieves high accuracy in joint angle measurements and repetition detection while offering increased robustness against occlusions. A comparative evaluation with RepNet, a state-of-the-art video-based repetition counting model, shows that the proposed method outperforms RepNet in both accuracy and computational efficiency, making it more suitable for real-time rehabilitation feedback. These findings highlight the potential of the proposed design to improve movement analysis reliability, optimize rehabilitation outcomes, and expand access to automated physical therapy assessment tools.

## Introduction

Human Pose Estimation (HPE) has become a critical tool for movement analysis, enabling detailed skeletal tracking through keypoint detection. Over the past decade, advancements in computer vision and artificial intelligence have significantly improved HPE capabilities, making it widely applicable in domains such as sports science, ergonomics, and healthcare^1^. In rehabilitation, HPE offers a non-invasive and scalable solution to monitor patient progress, enabling real-time analysis of joint movements and body posture^2,3^. Using machine learning (ML) algorithms, HPE systems can detect anatomical landmarks such as joints and limbs, allowing for biomechanical assessments, performance tracking, and injury prevention^4^. Despite these advantages, ML-based methods rely on large-scale annotated datasets, which introduce significant operational barriers, including high computational costs and challenges in domain adaptation^3,5^.

The integration of HPE into physical therapy represents a transformative advancement, particularly for home-based rehabilitation. Traditional rehabilitation practices rely on in-person assessments by physiotherapists, which can be costly and logistically challenging. Studies indicate that approximately 90% of rehabilitation exercises are performed at home without direct supervision^6^. Without real-time feedback, there is a risk that patients perform exercises incorrectly, leading to suboptimal recovery or even causing injury. Furthermore, physiotherapists often rely on qualitative visual assessments, which are subjective and prone to inaccuracies. Research suggests that, in low-speed activities, clinicians achieve only $12^\circ$ of accuracy in angular measurements, with a significantly lower accuracy for dynamic movements^7^. The lack of precise and quantitative tracking further complicates the evaluation of long-term progress^8^. HPE-based systems aim to bridge this gap by providing objective joint angle measurements, movement pattern analysis, and real-time exercise adherence feedback. By ensuring correct posture and movement execution, these systems have the potential to minimize errors in rehabilitation procedures and improve patient outcomes.

Computer-assisted rehabilitation technologies can be broadly categorized into wearable sensor-based and vision-based methods^9^. Sensor-based techniques utilize wearable devices, such as accelerometers and inertial measurement units, to capture motion data. Although these methods are effective, they require specialized hardware and may not be convenient for patients^10^. On the other hand, vision-based methods rely only on the data captured by standard RGB cameras, eliminating the need for additional equipment^11^. Vision-based methods can be further divided into two categories: keypoint-based pose estimation and activity recognition techniques. Keypoint-based pose estimation tracks individual joint positions^12^ while activity recognition classifies movements using spatial and temporal features^13^. Keypoint-based HPE has gained significant attention due to its ability to provide detailed movement analysis in a non-intrusive manner^14^. Recent studies have validated that such markerless systems can approach the accuracy of lab-based motion capture. For example, Liang et al.^15^ demonstrate that a 3D pose estimation system (using OpenPose/3DPoseNet) produce joint angle measurements within  $5^\circ$ of a gold-standard marker-based system, achieving high reliability in gait analysis. A similar study conducted by Aleksic et al.^16^ shows that a pose-based system could accurately measure vertical jump performance with negligible differences from a 3D motion capture reference. These results underscore that markerless HPE can attain clinical-level accuracy, supporting its use for quantitative rehabilitation monitoring.

Despite its promise, keypoint tracking in HPE presents several challenges that limit its effectiveness in physical therapy. One primary issue is occlusion, where overlapping body parts cause missing or inaccurate keypoint detection^17^. Additionally, pose estimation accuracy is influenced by external factors such as camera angle variations, lighting conditions, and subject-to-camera distance^18^. These challenges become even more pronounced during dynamic movements, leading to inconsistencies in keypoint visibility and erroneous movement analysis. Recent advancements, such as DAG (Data, Attention, Graph) augmentation, introduce instance-paste techniques and adaptive attention mechanisms^17^ to improve robustness in occluded scenarios. Similarly, unsupervised domain adaptation methods, such as the OR-POSE algorithm, refine pseudo-labels and integrate learned human pose priors to enhance prediction accuracy^19^.

A promising alternative to direct keypoint tracking is angular-based movement analysis, which evaluates joint angles and range of motion, rather than relying solely on individual keypoints^20^. This approach reduces sensitivity to occlusions and external disturbances, offering a more stable and consistent means of assessing movement quality^21^. Furthermore, normalization techniques such as bounding-box based standardization enhance consistency in pose data, ensuring accurate comparisons across different environments. Focusing on movement parameters such as range of motion, highest and lowest joint angles, and angular velocity, angular-based methods provide a higher level of abstraction for assessing rehabilitation exercises, making them more resilient to tracking errors.

To address the limitations of existing HPE methods in physical therapy, this study proposes an integrated approach that combines angular tracking with advanced keypoint normalization techniques. The proposed system leverages the MediaPipe framework for vision-based HPE, quantifying movement accuracy in real-time. Additionally, Dynamic Time Warping (DTW) and Normalized Cross-Correlation (NCC) are incorporated to enhance temporal alignment and movement similarity analysis. A fixed bounding-box strategy is applied across video frames to reduce noise and improve the reliability of keypoint comparisons. The proposed approach primarily aims to minimize the angular noise such as motion blur. Hence, the proposed system can be easily integrated with existing human pose estimation systems. In other words, our method is specifically designed to improve robustness in scenarios where detected keypoints contain positional noise—common in home-based rehabilitation setups—by leveraging angular relationships rather than absolute positions. This reduces sensitivity to scale, camera viewpoint, and jitter in the detected skeleton. These advancements contribute to an automated feedback mechanism, offering meaningful real-time insights to both clinicians and patients.

The proposed method is validated through a series of experiments comparing its effectiveness with conventional keypoint-based methods, including RepNet. Results demonstrate that our approach achieves better accuracy in joint angle measurements, reliable repetition counting, and improved robustness against occlusions, making it more suitable for real-time rehabilitation monitoring. By improving the reliability of movement analysis, this work aims to facilitate broader adoption of HPE-based solutions in both home-based and clinical rehabilitation settings, ultimately enhancing patient outcomes and reducing the burden on healthcare systems.

## Review of existing solutions

Real-time analysis of human motion, where feedback guides patients in performing rehabilitation exercises properly, is crucial in physical therapy. Various computational approaches have been explored to analyze movement, including vision-based techniques, angular-based movement analysis methods, and hybrid methodologies that integrate strategies for temporal alignments, such as DTW and NCC. While these methods have shown promise, they come with unique challenges and limitations, necessitating continued research to improve accuracy, robustness, and efficiency in rehabilitation settings.

Vision-based techniques primarily rely on RGB cameras to capture and analyze human movement non-invasively, making them cost-effective and suitable for real-time applications. Keypoint tracking, a core component of these methods, identifies specific anatomical landmarks to assess movement patterns^12^. However, these approaches are highly susceptible to occlusions, where overlapping body parts lead to missing or inaccurate keypoint predictions^17^. Moreover, variations in camera angles, lighting conditions, and subject-to-camera distances introduce inconsistencies in pose estimation^18^. To address these challenges, deep learning-based pose estimation frameworks such as OpenPose, PoseNet, and MediaPipe have been developed^4^. Although these models enhance real-time tracking, they remain limited in capturing complex movements due to their reliance on 2D projections, which lack depth information.

Recent advances in real-time pose estimation have further improved performance for rehabilitation applications. Dong and Du^1^ introduce an enhanced pose model based on YOLOv8 with a context coordination attention module, improving keypoint precision by  3% and recall by 4% compared to the base YOLOv8. The proposed CCAM-Person model outperforms Transformer-based approaches in both speed and accuracy while maintaining robustness in dense scenes, yielding a 4.7% higher accuracy in pose estimation under occlusions. Similarly, another study by Wang et al.^22^ shows that integrating RGB video with depth estimation helped mitigate occlusions in rehabilitation contexts. The proposed GaitPoseNet model achieves over 93% accuracy in in-bed pose estimation and processes pose outputs at more than 200 frames per second, emphasizing the trend towards lightweight yet high-performance models for home-based rehab. Furthermore, Zhang et al.^23^ introduce SR-POSE, a custom lightweight network that, when combined with a depth sensor, allows for real-time evaluation of rehabilitation exercises without wearable markers, making it both cost-effective and feasible for deployment in clinics or patient homes.

To overcome the limitations of keypoint tracking, angular-based methods focus on joint angles and range of motion instead of individual joint locations. This approach aligns more closely with clinical assessment criteria, as movement patterns are analyzed based on angular velocity, peak angles, and acceleration^21^. Unlike vision-based methods, angular-based techniques offer improved robustness against occlusions and external factors, making them more reliable for squats, knee extensions, and shoulder rotations^24^. Recent studies have demonstrated the feasibility of extracting joint angles from monocular 3D pose estimation models, improving the robustness of movement analysis. Mercadal-Baudart et al.^8^ introduce a pose estimation model trained on VICON motion capture data, achieving sub-$10^\circ$ accuracy in key rehabilitation metrics such as knee varus/valgus, hip flexion, and pelvic tilt. However, angular-based assessments still require precise keypoint normalization and tracking over time to ensure accurate motion analysis. Furthermore, Li et al.^25^ conduct a systematic review of resistance training for knee osteoarthritis (OA) patients. The authors state that although resistance training significantly improves gait velocity, it does not lead to notable improvements in knee adduction moment (KAM). This highlights the need to integrate additional biomechanical parameters beyond joint moments for comprehensive rehabilitation assessments.

Temporal variations in exercise execution present further challenges in movement analysis, as patients often perform exercises at different speeds, making direct frame-by-frame comparisons unreliable. DTW has been widely adopted to address this issue by dynamically aligning movement sequences of varying lengths, ensuring comparability regardless of execution speed^26^. Moreover, NCC measures the spatial similarity between movement sequences by assessing displacement patterns, further enhancing robustness in tracking deviations from ideal motion trajectories^27^. The integration of DTW and NCC in rehabilitation systems significantly improves the accuracy of movement assessments, but computational efficiency remains a concern, especially for real-time applications.

Accurate repetition counting is another crucial aspect of rehabilitation monitoring. RepNet, a neural network designed for repetition counting, employs temporal convolutional networks to detect periodic motion patterns^9^. However, its applicability in physical therapy is limited due to accuracy constraints and high computational demands. Alternative methods based on joint angle trajectories and peak detection have shown superior accuracy by focusing on movement peaks and troughs, which are more clinically relevant for tracking patient performance^28^. Angular-based movement analysis offers a more scalable solution, reducing dependence on deep learning models that require extensive data for training and computational resources.

The MediaPipe framework has gained traction in human pose estimation due to its efficiency in real-time pose tracking. Using deep learning models and normalization techniques, MediaPipe ensures standardized pose data across different camera angles and subject positions^29^. Keypoint normalization plays a vital role in mitigating perspective distortions, with techniques such as bounding-box normalization and relative coordinate transformation improving consistency across frames^30^. These improvements make MediaPipe an effective tool for rehabilitation motion analysis, particularly when combined with angular-based assessment techniques.

Despite these massive advancements, several challenges persist in ensuring the accuracy and reliability of systems designed for motion analysis in physical therapy. Firstly, occlusion remains a significant issue in keypoint tracking, particularly in multi-limb movements, where overlapping body parts cause incomplete or inaccurate data. Angular-based approaches mitigate this limitation by focusing on joint motion rather than absolute keypoint positions. Secondly, variations in camera angles and subject-to-camera distances introduce further inconsistencies, necessitating robust normalization techniques such as bounding-box standardization and camera-invariant feature extraction. Thirdly, temporal misalignment of movement sequences complicates real-time analysis, with DTW offering a viable solution at the cost of computational complexity. In addition, the high processing demands of deep learning-based models, such as RepNet, limit their practicality for real-time rehabilitation monitoring, emphasizing the need for optimized machine learning models and heuristic-based repetition detection methods. Finally, the lack of personalized feedback mechanisms remains a critical limitation, as current systems struggle to adapt to the needs of the individual patient. This underscores the importance of developing adaptive algorithms that dynamically adjust feedback based on patient progress, ensuring a more tailored rehabilitation experience. Future advancements in movement analysis for physical therapy will likely emerge from hybrid methodologies that integrate keypoint tracking, angular-based movement analysis, and advanced normalization techniques, significantly enhancing the reliability and effectiveness of rehabilitation assessment systems.

## Proposed methodology

**Figure 1:**
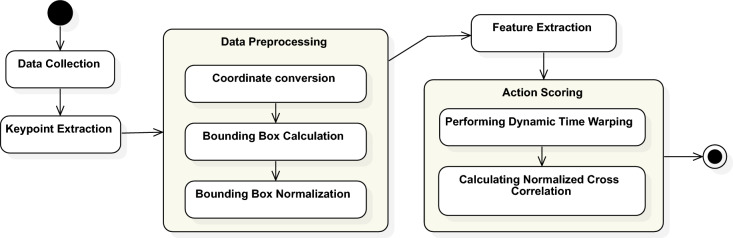
Steps of the proposed methodology: The process begins with data collection and keypoint extraction, followed by preprocessing steps including coordinate conversion, bounding box calculation, and normalization. Feature extraction is then performed to generate descriptors, which are compared using Dynamic Time Warping (DTW) and Normalized Cross-Correlation (NCC) for action scoring.

This study presents a computer vision-based system for evaluating physical therapy exercises using pose estimation and movement analysis. The system integrates spatial and temporal analysis techniques, primarily focusing on a novel angle-based method for scoring movement quality and range of motion. Figure 1 shows the main steps and substeps involved in each step of the proposed methodology. The methodology begins with data collection, involving recording high-quality rehabilitation exercise videos under controlled conditions. Keypoint extraction uses MediaPipe Pose, which detects 33 anatomical landmarks per frame and produces structured pose data. In the preprocessing stage, keypoints are converted from normalized coordinates to pixel values, aligned using bounding boxes, and normalized to a consistent reference frame to ensure comparability across frames and subjects. This normalized data extracts joint-level biomechanical features, such as joint angles, range of motion (ROM), and movement velocity. These features form the basis of the proposed angular-based scoring method, which evaluates how closely a subject’s movement aligns with clinically demonstrated reference motions. The system employs an angle-based repetition counting approach that identifies peaks and troughs in joint angle trajectories to segment repeated movements. To evaluate the effectiveness of this method, we compare it against RepNet, a state-of-the-art deep learning-based repetition counting model.

The following sections describe each methodology stage, including the proposed scoring approach and supporting components, in more detail.

### Data collection

The exercise data were collected using high-quality video recordings of prescribed physical therapy exercises performed under controlled conditions. Instead of involving patients, the data consists of professionally filmed demonstrations by the research team. High-resolution digital cameras, capable of recording at 60 frames per second, were positioned to capture both frontal and side views, ensuring comprehensive visibility of joint movements.

A total of six healthy adult participants, aged between 20 and 35 years, were recruited to perform the exercises. Each participant completed multiple movement demonstrations, including both standard and intentionally altered forms, to introduce natural variability for system validation. All exercises were recorded using a single high-resolution camera, positioned to capture either a frontal or lateral view depending on the intended perspective. No multi-camera synchronization or fusion was used; each video represents a distinct viewpoint recorded independently.

To minimize external variations that could impact pose estimation accuracy, the recording environment was standardized with evenly distributed diffused LED lighting to reduce shadows and enhance the clarity of anatomical landmarks. A diverse range of physical profiles was represented in the demonstration videos, including different body types and mobility levels. Additionally, benchmark data were obtained by having trained physiotherapists and rehabilitation specialists perform the same exercises under identical recording conditions. Each exercise video is synchronized using timestamps to ensure consistency in movement analysis and enable accurate temporal alignment. Exercises are repeated multiple times, adhering to standardized rehabilitation protocols to capture a representative dataset. Given the limitations of traditional keypoint tracking methods, which may suffer from occlusion and inaccuracies due to variations in camera angles and distances, the collected data is designed to support both keypoint tracking-based and angular-based movement analysis.

Ethical principles regarding data collection were carefully considered throughout the study. All procedures involved healthy adult volunteers who were also members of the research team at Augmented Vision Ltd performing standard physical therapy exercises for demonstration purposes. Although no patients or clinical interventions were involved, all experimental protocols were reviewed and approved by Augmented Vision Ltd’s internal ethics review process. All experiments were performed in accordance with relevant institutional and regulatory guidelines. Written informed consent was obtained from all participants for both participation and publication of anonymized movement data and visual materials. All personal identifiers were removed, maintaining strict anonymity and compliance with ethical research standards.

This rigorous data collection strategy provides a robust foundation for developing and evaluating an action-scoring algorithm capable of accurately assessing rehabilitation exercises.

### Keypoint extraction

After data collection, pose estimation was conducted using the MediaPipe Pose framework^31^. MediaPipe detects 33 anatomical landmarks from each video frame, including key joints and limbs. This process generates structured motion data essential for quantitative movement analysis.

For each frame, MediaPipe provides normalized $(x, y, z)$ coordinates relative to the frame dimensions, along with visibility confidence scores for each keypoint. These outputs are stored in a structured JSON format, which serves as the foundation for all subsequent stages of preprocessing, feature extraction, and scoring.

The structure of the keypoint data is summarized in Table 1, and includes metadata such as frame count, resolution, and frame rate, facilitating accurate and scalable motion analysis.

**Table 1:** Structure of the JSON output generated during keypoint extraction.

Item	Description
Timeline	Keypoint data across all frames
TotalNumberOfFrames	Number of frames with valid keypoints
Items	Per-frame keypoint coordinates
Width	Frame width (in pixels)
Height	Frame height (in pixels)
AverageFPS	Video frame rate (frames per second)

The data includes frame-wise keypoint coordinates, video resolution, and frame rate, essential for accurate movement analysis

MediaPipe was selected for this study due to its computational efficiency, real-time processing capability, and ease of deployment on resource-constrained devices^4^. While other high-accuracy pose estimation models, such as HRNet or advanced OpenPose variants, demonstrate improved precision on benchmark datasets, they typically require dedicated GPU hardware and introduce latency that can hinder real-time feedback in home-based rehabilitation contexts^32^. Moreover, recent studies indicate that when joint angles and derived kinematic features are the primary outputs, angular-based methods are less sensitive to minor keypoint localization errors, mainly when supported by normalization and visibility filtering^8^. Given the practical trade-off between computational overhead and estimation precision, MediaPipe was deemed appropriate for the goals of this study, which include real-time feedback and accessibility in home rehabilitation environments.

### Data preprocessing

Data preprocessing is a critical step that refines the raw keypoint data obtained from pose estimation to ensure consistency and comparability across video recordings. Although Media Pipe provides normalized keypoint coordinates, variations in subject position, body proportions, and camera setup can still introduce inconsistencies that affect downstream analysis.

To address these issues, the preprocessing pipeline applies three key operations:

1. Conversion of MediaPipe’s normalized keypoint coordinates to absolute pixel values,
2. Bounding box calculation to define a stable region of interest, and
3. Normalization of keypoints relative to a fixed bounding box for spatial consistency.

These operations stabilize the data across frames and subjects by aligning movements to a common spatial reference frame. The resulting preprocessed data provides a reliable foundation for extracting kinematic features and performing accurate movement comparisons.

#### Coordinate conversion

Keypoint normalization is essential to ensure spatial consistency across different video recordings, regardless of resolution or subject positioning. Each detected keypoint is represented by a pair of normalized coordinates $(x, y)$, expressed as the video frame’s width and height proportions. This format ensures that keypoints maintain a consistent relative position across videos with different resolutions or subject distances from the camera.

**Figure 2:**
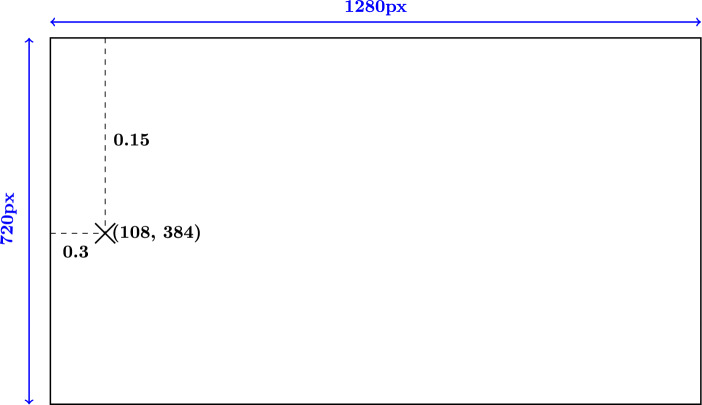
Example of pose detection output. Each detected keypoint includes normalized coordinates and their corresponding absolute pixel positions, both relative to the frame dimensions.

The extracted keypoint data is stored in a structured JSON format. For each frame, this format contains normalized keypoint coordinates, frame dimensions (width and height in pixels), total number of frames, frame rate (FPS), and visibility confidence scores for each keypoint. These values provide a scalable representation of human motion over time. Figure 2 illustrates an example of a pose detection output, where each detected keypoint is assigned a coordinate based on the dimensions of the frame.

As we can see, the normalized coordinates (e.g., $x = 0.15, y = 0.3$) are mapped to absolute pixel values (e.g., $X = 108, Y = 384$) using the known frame resolution (e.g., $1280\times 720$). This transformation is performed using the following equations:

1 $$\begin{aligned} \begin{aligned} X&= x \times \text {Width}_{\text {frame}} \\ Y&= y \times \text {Height}_{\text {frame}} \end{aligned} \end{aligned}$$

Where, $x, y$ are normalized horizontal and vertical coordinates, $\text {Width}_{\text {frame}}, \text {Height}_{\text {frame}}$ represent the actual frame dimensions in pixels, and $X, Y$ are absolute pixel locations.

This conversion is necessary for visualizations, overlaying keypoints on frames, and computing derived features such as joint angles and movement trajectories. It ensures that keypoint data remains spatially accurate and consistent across the dataset, forming a reliable basis for subsequent feature extraction and movement scoring.

#### Bounding box calculation

Accurate movement analysis requires identifying a stable region of interest for each subject. To achieve this, a bounding box is computed for every frame to encapsulate all visible keypoints. This bounding box helps localize the subject and prepare the data for frame-to-frame comparison. The bounding box is calculated by identifying the extreme horizontal and vertical positions of the detected keypoints, with a predefined buffer applied to ensure that limb movements near the edges are not truncated:

2 $$\begin{aligned} \text {Bounding Box} = \begin{bmatrix} \min (X_i) - \text {buffer}, & \max (X_i) + \text {buffer} \\ \min (Y_i) - \text {buffer}, & \max (Y_i) + \text {buffer} \end{bmatrix} \end{aligned}$$

where $X_i$ and $Y_i$ represent the pixel coordinates of all keypoints in a given frame, and $\text {buffer}$ is an additional margin to prevent keypoints from being cut off.

While frame-by-frame bounding boxes can be effective, they introduce instability during analysis. Minor variations in keypoint detection may cause the bounding box to jitter, even when the subject is relatively static. This artificial motion can compromise the reliability of movement tracking. To address this, the system computes a fixed maximum bounding box based on the largest observed height and width across the entire video. This ensures consistent spatial framing across all frames, minimizing noise and enhancing the temporal stability of pose tracking.

**Figure 3:**
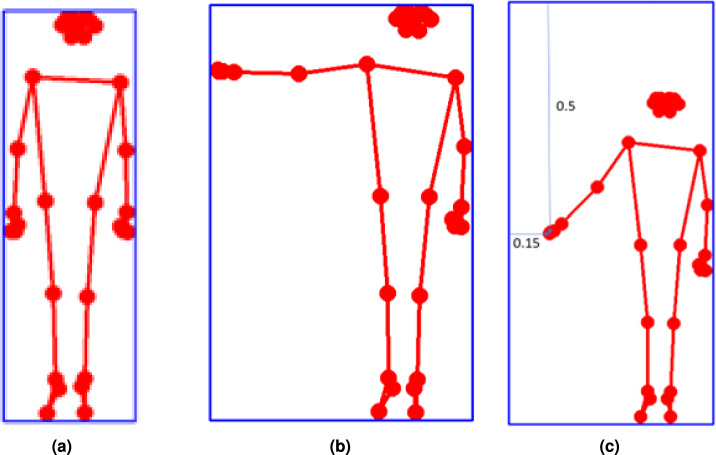
(**a**) Computation of the bounding box for a single frame, encapsulating detected keypoints for movement tracking. (**b**) Comparison of dynamic bounding box resizing (left) versus fixed maximum bounding box (right), demonstrating improved stability for movement analysis. (**c**) Normalization of keypoint values relative to the fixed bounding box, ensuring consistency in movement tracking despite variations in subject placement or camera position.

Figure 3a,b illustrates this process and the difference between dynamic and fixed bounding box strategies.

#### Bounding box-based normalization

Once the fixed bounding box is established, keypoint coordinates are normalized relative to this reference frame. This transformation removes the influence of camera framing, subject placement, and screen size, enabling consistent comparison across frames and subjects.

The normalized coordinates are computed as:

3 $$\begin{aligned} X_{\text {final}} = \frac{(X - \text {Bounding Box}_{\min X})}{\text {Width of Max Bounding Box}} \end{aligned}$$

4 $$\begin{aligned} Y_{\text {final}} = \frac{(Y - \text {Bounding Box}_{\min Y})}{\text {Height of Max Bounding Box}} \end{aligned}$$

Where $X$ and $Y$ are the absolute pixel coordinates of a keypoint and $\text {Bounding Box}_{\min X}$, $\text {Bounding Box}_{\min Y}$: top-left corner of the fixed bounding box. The $\text {Width of Max Bounding Box}$, $\text {Height of Max Bounding Box}$ represents the dimensions of the fixed bounding box.

This spatial normalization step ensures that movements are analyzed within a consistent coordinate system, independent of external recording conditions. It significantly improves the accuracy of temporal and spatial comparisons in techniques such as DTW and NCC.

Figure 3c demonstrates the final stage of normalization applied to keypoint coordinates, following bounding box stabilization. Overall, this process enhances the reliability and repeatability of movement features, laying a stable foundation for robust rehabilitation scoring and feedback.

### Feature extraction

**Figure 4:**
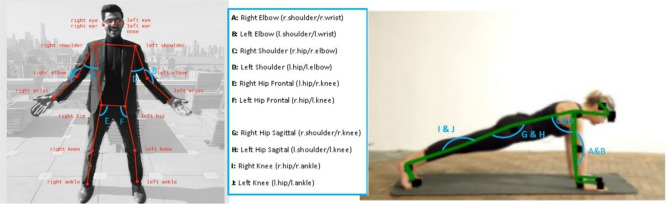
Tracked joint angles used for movement analysis in physical therapy. The left image depicts labeled keypoints used for pose estimation, while the right image visualizes the key angles (**A**–**J**) extracted to quantify range of motion, movement smoothness, and exercise accuracy^33^.

Following the preprocessing stage, the next crucial step is feature extraction. Feature extraction aims to identify and quantify essential aspects of movements captured in the video data, which are necessary to accurately and objectively compare patient and demonstration videos. Specifically, it involves deriving various kinematic features, such as joint angles, ROM, and movement velocities, from the normalized keypoints, which are critical for evaluating the quality of movement. Figure 4 illustrates an example of the key angles used in movement analysis, highlighting the anatomical regions considered to track the range of motion, the highest and lowest angles, and the quality of movement.

Joint angles are calculated using the coordinates of keypoints associated with specific joints. For instance, we use the coordinates of the shoulder, elbow, and wrist joints to compute the elbow angle. Angle $\theta$ is determined using the cosine rule:

5 $$\begin{aligned} \cos (\theta ) = \frac{\textbf{a} \cdot \textbf{b}}{\Vert \textbf{a}\Vert \Vert \textbf{b}\Vert } \end{aligned}$$

where $\textbf{a}$ and $\textbf{b}$ are vectors formed by the coordinates of the joints:

6 $$\begin{aligned} \textbf{a} = (x_{\text {shoulder}} - x_{\text {elbow}}, y_{\text {shoulder}} - y_{\text {elbow}}) \end{aligned}$$

7 $$\begin{aligned} \textbf{b} = (x_{\text {wrist}} - x_{\text {elbow}}, y_{\text {wrist}} - y_{\text {elbow}}) \end{aligned}$$

The angle $\theta$ can also be computed using the dot product formula as:

8 $$\begin{aligned} \theta = \arccos \left( \frac{\textbf{a} \cdot \textbf{b}}{\Vert \textbf{a}\Vert \Vert \textbf{b}\Vert }\right) \end{aligned}$$

This calculation is performed for each joint on all frames. Furthermore, the ROM for each joint is determined by measuring the difference between the maximum and minimum joint angles observed during an exercise:

9 $$\begin{aligned} \text {ROM} = \theta _{\text {max}} - \theta _{\text {min}} \end{aligned}$$

where $\theta _{\text {max}}$ and $\theta _{\text {min}}$ are the maximum and minimum joint angles recorded. Finally, the velocity, which describes the rate of movement, is computed by differentiating the keypoint positions over time:

10 $$\begin{aligned} \textbf{v}(t) = \frac{\textbf{p}(t+1) - \textbf{p}(t-1)}{2 \Delta t} \end{aligned}$$

where $\textbf{p}(t)$ is the position of the keypoint in frame *t* and $\Delta t$ is the time interval between frames. This central difference method smooths the velocity estimate, reducing noise.

The extracted movement features were normalized to ensure consistency across subjects. This normalization process compensates for body size, orientation, and movement amplitude variations, allowing for reliable inter-subject comparison. The process consists of two key steps: mean subtraction and amplitude normalization. First, to eliminate positional offsets, the mean of each feature is subtracted from the actual values using the formula:

11 $$\begin{aligned} X' = X - \mu _X \end{aligned}$$

where $\mu _X$ is the mean of feature *X*. This centers the signal around zero.

Next, to ensure consistency in the magnitude of movements, each feature is scaled to unit variance:

12 $$\begin{aligned} X'' = \frac{X'}{\sigma _X} \end{aligned}$$

Where $\sigma _X$ is the standard deviation of the feature *X*. This allows for fair movement comparisons.

By applying mean subtraction and amplitude normalization to each extracted feature, we ensure that the data is standardized, making the signals from the patient and the demonstration directly comparable.

### Action scoring algorithm

**Figure 5:**
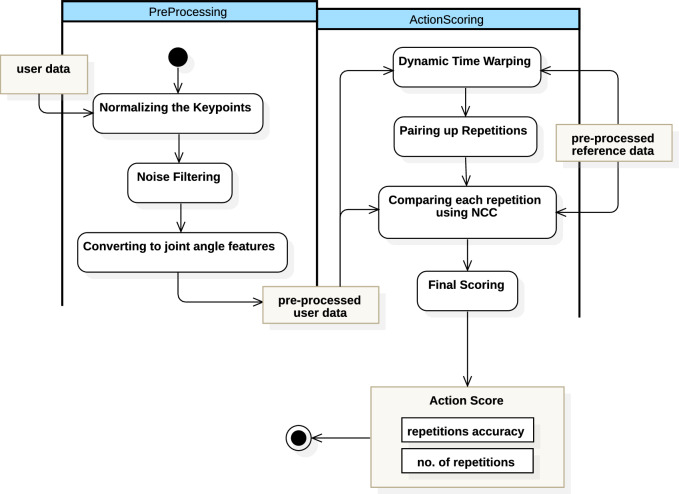
Overview of the action scoring pipeline. User and reference videos are processed through pose detection, keypoint normalization, noise filtering, and conversion to joint-angle features. Dynamic Time Warping (DTW) aligns both sequences, and paired repetitions are compared using Normalized Cross-Correlation (NCC). The combined similarity and repetition accuracy yield the final *Action Score*.

The action scoring algorithm evaluates movement accuracy by comparing detected keypoints with pre-stored ideal movement models. It is important to note that the ideal model serves as a clinically validated reference rather than an independent ground truth. As illustrated in Fig. 5, this process involves parsing JSON data received from the front end, loading the ideal reference data, preprocessing the user’s movement data, and performing a comparison. The comparison uses DTW and NCC to measure temporal and spatial alignment. RepNet is used as a benchmark model for evaluating the proposed angle-based method. Each of these methods is detailed in the following subsections.

The system begins with two types of input data: a user exercise video and a reference demonstration video. Both videos are processed using MediaPipe, which detects the human body’s keypoints frame by frame and saves them as JSON files. These files contain the *x*–*y* coordinates of each joint over time, forming time-series data that represent the person’s movements. As shown in Fig. 5, this raw data provides the foundation for subsequent processing.

In the preprocessing stage, both datasets (user and reference) are cleaned and standardized. This involves normalizing the keypoints within a bounding box to remove scale and camera-position effects, filtering out noise, and then converting the keypoints into joint angle features such as ROM and angular velocity. This angular representation minimizes the influence of occlusion, camera distance, and subject size, offering a clinically meaningful abstraction of motion. After this step, both datasets are transformed into a comparable format describing the motion of each joint over time.

Next, the system identifies repetition frames by detecting peaks and troughs in the joint angle trajectories, determining the start and end points of each movement cycle. This segmentation enables repetition-by-repetition comparison between user and reference data. To account for timing differences (e.g., if the user performs faster or slower than the demonstration), the user’s processed data are aligned with the reference using DTW. DTW minimizes temporal discrepancies, ensuring that equivalent phases of motion are compared.

Once the temporal alignment is achieved, NCC is applied to measure the similarity between the motion curves of each repetition. NCC quantifies how closely the user’s joint trajectories match the reference trajectory, producing similarity values between $-1$ and 1. These values are aggregated across all repetitions and integrated with the repetition-counting accuracy to compute the final Action Score. This composite score reflects both how many repetitions were correctly performed and how accurately each movement matched the reference model.

Through this integrated pipeline—from pose extraction to angular feature comparison—the algorithm systematically accounts for temporal, spatial, and kinematic variations. This ensures fair, interpretable, and clinically relevant assessment of user performance, aligning automated evaluations with physiotherapist standards.

#### Dynamic time warping

Dynamic Time Warping (DTW) is a technique for measuring the similarity between two temporal sequences that may vary in speed. It aligns sequences by stretching or compressing the time axis to minimize the distance between corresponding points, making it particularly effective for comparing movements at different speeds. Mathematically, the DTW distance between two sequences, $P$ (patient’s movement) and $D$ (demonstration sequence), is computed as:

13 $$\begin{aligned} DTW(P, D) = \sqrt{\sum _{i=1}^{n} (P(i) - D(i))^2} \end{aligned}$$

where $P(i)$ and $D(i)$ represent the values of the sequences at index $i$, and $n$ is the length of the sequences. DTW minimizes this distance by allowing non-linear alignments between the sequences.

We use the dtaidistance Python library to implement DTW, which efficiently computes the DTW distance and provides alignment visualizations for movement analysis.

#### Normalized cross-correlation

Normalized Cross-Correlation (NCC) is a statistical method used to measure the similarity between two signals or datasets. It is particularly useful for comparing signals with different amplitudes or varying lengths. The NCC produces a value between $-1$ and $1$, where:

- $1$ indicates a perfect positive correlation,
- $-1$ indicates a perfect negative correlation, and
- $0$ indicates no correlation.

In this context, NCC is used to compare individual repetitions of the patient’s movements with those in the demonstration video. Calculating normalized cross-correlation involves several steps: preparing the signals, computing the cross-correlation, finding the maximum value, and storing the results for each repetition.

1. Preparing the Signals First, the normalized features for each repetition from the patient’s and the demonstration’s videos are arranged into one-dimensional signals, denoted as $X$ and $Y$. These signals must be of equal length and correspond to the same time frames. Let $X(t)$ and $Y(t)$ represent the normalized features at time $t$.
2. Computing the Cross-Correlation The cross-correlation between the two signals $X(t)$ and $Y(t + \tau )$ at a time lag $\tau$ is computed as:
  14 $$\begin{aligned} R_{XY}(\tau ) = \frac{\sum _{t} (X(t) - \mu _X) (Y(t + \tau ) - \mu _Y)}{\sigma _X \sigma _Y} \end{aligned}$$
  where:
  $R_{XY}(\tau )$ is the cross-correlation at time lag $\tau$,
  $X(t)$ and $Y(t + \tau )$ are the values of the signals at times $t$ and $t + \tau$, respectively,
  $\mu _X$ and $\mu _Y$ are the mean values of signals $X$ and $Y$, and
  $\sigma _X$ and $\sigma _Y$ are the standard deviations of signals $X$ and $Y$.
3. Finding the Maximum Cross-Correlation Value The highest degree of similarity between the patient’s repetition and the demonstration repetition is determined by finding the maximum value of $R_{XY}(\tau )$ across all possible time lags:
  15 $$\begin{aligned} R_{\text {max}} = \max _{\tau } R_{XY}(\tau ) \end{aligned}$$
4. Storing the Results For each repetition, the maximum cross-correlation value $R_{\text {max}}$ is stored. These values are then used to assess the similarity between the patient’s movements and the demonstration. Finally, the overall similarity score $S$ is obtained by averaging the maximum cross-correlation values across all repetitions:
  16 $$\begin{aligned} S = \frac{1}{N} \sum _{i=1}^{N} R_{\text {max}}^{(i)} \end{aligned}$$
  where $N$ is the total number of repetitions analyzed, and $R_{\text {max}}^{(i)}$ represents the maximum cross-correlation value for the $i$-th repetition.

Following these steps, we obtain a comprehensive measure of how closely the patient’s movements align with the demonstration, providing an objective basis for the action scoring system.

#### Comparison with RepNet

RepNet is a deep learning-based repetition counting model that employs a Temporal Convolutional Network (TCN) to detect periodic motion patterns in videos. It analyzes temporal dependencies in frame sequences to identify repeated movements without requiring explicit joint tracking or pose estimation. The model outputs a scalar count representing the total number of repetitions detected in a given video segment. To use RepNet for benchmarking, we adopted an open-source implementation of the model. The input video data was preprocessed to meet RepNet’s requirements. Each frame was resized to the standard resolution expected by the model, and pixel values were normalized across all channels. The video sequences were also adjusted to maintain a consistent frame rate and duration when necessary.

Once preprocessed, the entire video sequence was passed through RepNet, which processed the frames and directly produced an estimated repetition count. RepNet does not segment the video into individual repetitions nor provide joint-level information. Therefore, its output was used solely for comparison with the angular-based movement analysis method described earlier. By applying RepNet to the same set of exercise videos used in our proposed system, we established a consistent evaluation framework for assessing repetition counting performance across different methods.

## Results

This section presents the results of the action scoring algorithm in our proposed model, emphasizing the accuracy and reliability of the system in evaluating exercise performance. The remainder of this section offers statistical analyses, visual representations of the data, and comparisons between automated scores and expert evaluations.

### Experimental setup

To validate the effectiveness of the action scoring algorithm, a series of experiments is conducted using video data from both patients and professional demonstrations. The input dataset comprises diverse participants performing a set of predefined exercises. The primary metrics for evaluation include joint angles, range of motion, and movement velocities, as extracted and normalized in the previous stages.

### Keypoint detection accuracy

The performance of the action scoring algorithm is evaluated using a dataset of video recordings featuring various prescribed physical therapy exercises. The keypoint detection accuracy is measured by comparing the detected keypoints against manually annotated ground truth data. The keypoint detection accuracy of the proposed system is found to be highly reliable, with an average deviation of less than 10% from manually annotated ground truth data as depicted in Table 2. This high level of precision is critical for accurate movement analysis and feedback in physical therapy. Furthermore, the consistency in keypoint detection across various exercises demonstrates the robustness of the MediaPipe framework and the preprocessing techniques applied, such as keypoint normalization and bounding box adjustments. In summary, the experimental results confirm the ability of the system to provide detailed and reliable data for further movement analysis and feedback.

### Accuracy of joint angle measurements

The accuracy of the joint angle measurements is assessed by comparing automated calculations from the action scoring system against expert clinician evaluations. Clinicians manually measured joint angles by reviewing the exercise videos frame by frame, using anatomical landmarks to estimate the angle between relevant limbs (e.g., shoulder-elbow-wrist for elbow flexion) based on established clinical goniometric techniques. These manual values served as a reference standard, as such visual angle estimations are routinely used in clinical practice to assess ROM and are widely accepted for their reliability under controlled conditions^7,34^. The agreement between the automated and manual values was quantified using mean absolute error (MAE).

**Figure 6:**
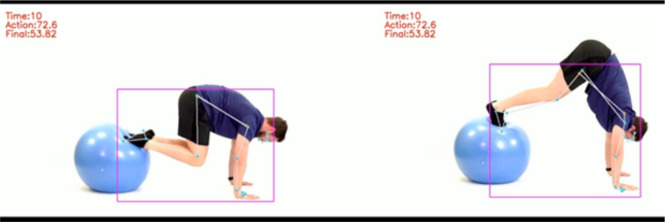
Comparison of detected keypoints for subject (right) versus demonstration (left), showcasing deviations in joint angles.

Figure 6 provides a visual representation of the detected keypoints from both the subject and the demonstration, highlighting misalignment in key joints.

**Table 2:** Keypoint detection accuracy for different exercise types.

Exercise type	GT keypoints	Detected keypoints	Deviation (%)
Active shoulder abduction	1000	920	8
Bodyweight deep squat	1000	910	9
Bending elbow	1000	930	7
Straight leg raise	1000	920	8

The ground truth (GT) keypoints represent the expected number of detected points, while the deviation percentage quantifies the difference between detected and GT keypoints

To provide a comprehensive assessment, Table 3 presents a merged comparison of joint angles from both the demonstration and the subject data. Table 3 consolidates previously recorded measurements, ensuring a structured evaluation of the range, highest, and lowest angles observed during movement execution.

The values presented in Table 3 are based on manually annotated and averaged automated measurements. All values were rounded to the nearest integer to match clinical reporting conventions and enhance readability. These figures serve as high-level summary comparisons rather than precise frame-level outputs.

**Table 3:** Rounded comparison of joint angles between demonstration and subject recordings.

Joint	Range ($\circ$)	Demo max ($\circ$)	Demo min ($\circ$)	Subject Mmax ($\circ$)	Subject min ($\circ$)
Right elbow	0	180	180	180	180
Left elbow	0	180	180	180	180
Right shoulder	40	130	90	150	90
Left shoulder	40	130	90	150	90
Right Hip Frontal	0	0	0	0	0
Left hip frontal	0	0	0	0	0
Right hip sagittal	135	180	45	180	90
Left hip sagittal	135	180	45	180	90
Right knee	120	170	50	170	160
Left knee	120	170	50	170	160

Values reflect the range, maximum, and minimum angles for each joint, manually annotated and averaged from automated outputs, and rounded to match clinical reporting formats.

Table 4 further evaluates the differences between the demonstration and the movements of the subject, highlighting key deviations and necessary adjustments. This allows for structured feedback based on variations in the range, highest, and lowest angles recorded. The results indicate key discrepancies in the execution of the movement. In particular, the subject’s shoulders extended $20^\circ$ beyond the demonstration, suggesting possible overcompensation for restricted lower body movement. In addition, significant flexion deficits were observed in the hip and knee joints, with the subject’s right and left hip flexion angles being $45^\circ$ lower than the demonstration, while the knee flexion angles showed a $110^\circ$ deficit. These variations suggest insufficient depth in the execution of the movement. The feedback in Table 4 is derived directly from the comparison data. The results emphasize that major adjustments are required in hip and knee flexion to align the subject’s movements with the ideal demonstration. These findings further validate the importance of using both manual and automated assessments for accurate movement evaluation.

As with Table 3, the values shown in Table 4 are based on post-processed angular metrics and are reported as rounded integers to align with clinical feedback formats. The table highlights movement trends and areas needing correction rather than exact numerical precision.

**Table 4:** Rounded deviations in joint angles between subject and demonstration with corresponding feedback.

Joint	Range diff ($\circ$)	Max diff ($\circ$)	Min diff ($\circ$)	Feedback
Right elbow	0	0	0	No significant deviation
Left elbow	0	0	0	No significant deviation
Right shoulder	$-$20	$-$20	0	Right shoulder extending $20\circ$ more than demo
Left shoulder	$-$20	$-$20	0	Left shoulder extending $20\circ$ more than demo
Right hip frontal	0	0	0	No deviation
Left hip frontal	0	0	0	No deviation
Right hip sagittal	45	0	$-$45	Right hip flexing $45\circ$ less than demo
Left hip sagittal	45	0	$-$45	Left hip flexing $45\circ$ less than demo
Right knee	110	0	$-$110	Right knee flexing $110\circ$ less than demo
Left knee	110	0	$-$110	Left knee flexing $110\circ$ less than demo

Comparison of maximum and minimum joint angles between the user and the ideal model also provides insightful data, allowing for a more refined analysis of deviations, considering both the range and extreme values of joint movements as presented in Table 5. In other words, including maximum and minimum joint angles significantly enhances the evaluation of movement accuracy. Notably, the right elbow exhibited a $20.54^\circ$ increase in maximum flexion compared to the ideal model, and the right hip reached 18.43° beyond the expected range. These deviations provide further evidence of movement inconsistencies. Furthermore, differences in minimum joint angles indicate variations in movement depth, such as the left hip flexing 9.28° less than expected.

**Table 5:** Comparison of maximum and minimum joint angles between user and ideal model.

Joint	User max ($\circ$)	Ideal max ($\circ$)	Max diff ($\circ$)	User min ($\circ$)	Ideal min ($\circ$)	Min diff ($\circ$)
Left shoulder	42.17	47.03	$-$4.86	28.94	36.89	$-$7.95
Right shoulder	176.18	165.70	10.48	29.17	26.01	3.17
Left elbow	42.17	47.03	$-$4.86	28.94	36.89	$-$7.95
Right elbow	206.30	185.75	20.54	143.41	118.44	24.97
Left hip	175.90	179.61	$-$3.71	154.33	163.61	$-$9.28
Right hip	197.81	179.37	18.43	171.68	160.22	11.45
Left knee	181.82	183.71	$-$1.88	169.09	163.31	5.78
Right knee	181.22	190.21	$-$9.00	167.02	166.48	0.53

By integrating this additional layer of analysis, the evaluation becomes more comprehensive, ensuring that both significant deviations and minor variations are taken into account when assessing movement similarity.

### Dynamic time warping and normalized cross correlation

The action scoring algorithm uses DTW and NCC to compare user movements against ideal models. DTW effectively aligns the temporal sequences of the users and ideal movements, while NCC measures the similarity between the two sequences. DTW and NCC provide robust movement analysis, enabling detailed comparison and accurate feedback on exercise performance. The quantitative measures from these techniques indicate a less than 10% deviation from the ideal models, underscoring the algorithm’s effectiveness in performance assessment as shown in Table 6.

The NCC values provide a robust similarity measure between patient movements and demonstration videos. The average NCC score across all repetitions for each exercise is above 0.85, indicating a high level of correlation. This suggests that the patient’s movements closely mirrors the ideal model, validating the effectiveness of the NCC approach in capturing the precision of movement.

**Table 6:** Effectiveness of DTW and NCC in movement analysis.

Metric	Value
Average deviation from ideal model (%)	< 10%
Alignment accuracy (DTW)	> 90%
Sequence similarity (NCC)	> 0.85

The table presents the average deviation from the ideal movement model, alignment accuracy using DTW, and sequence similarity based on NCC

### Repetition counting precision

The repetition counting mechanism is evaluated based on its ability to accurately identify the start and end points of each exercise repetition. It utilizes angular calculations to detect peaks and troughs in joint movements, effectively segmenting individual repetitions from continuous motion data.

To assess accuracy, the following error formula is used:

17 $$\begin{aligned} \text {Error (\%)} = \left| \frac{\text {Detected Repetitions} - \text {Actual Repetitions}}{\text {Actual Repetitions}} \right| \times 100 \end{aligned}$$

This metric quantifies the percentage deviation between the system’s detected count and the actual number of repetitions performed.

Table 7 presents the precision of repetition counting across four common physiotherapy exercises. The system demonstrates low detection error, with an average marginal error of approximately 7.5%. The lowest observed error was 5% for the Bending Elbow exercise, while the highest was 10% for Active Shoulder Abduction. These results indicate the system’s robustness in identifying repetition boundaries with reasonable accuracy. However, the total number of actual repetitions across all exercises in this evaluation is fewer than 100. As such, even a single miscounted repetition can disproportionately affect the error percentage. For instance, one incorrect count in a 10-repetition set results in a 10% error. While the results are encouraging, this small sample size introduces variability, and the reported metrics should be interpreted as preliminary indicators rather than definitive performance benchmarks. We acknowledge this limitation and plan to expand the evaluation dataset in future work.

**Table 7:** Repetition counting precision across different exercise types.

Exercise type	Actual reps	Detected reps	Error (%)
Active shoulder abduction	10	9	10.0
Bodyweight deep squat	15	14	6.7
Bending elbow	20	19	5.0
Straight leg raise	12	11	8.3

The table presents the actual number of repetitions performed, the number of repetitions detected by the system, and the corresponding detection error percentage.

In addition to repetition count accuracy, the system was also evaluated on biomechanical performance across segmented repetitions. Table 8 presents the average range of motion (ROM) and angular velocity (mean ± standard deviation) across repetitions for selected exercises. These metrics were computed using knee joint angles segmented between repetition boundaries.

**Table 8:** Per-repetition ROM and velocity variability across selected exercises.

Exercise	Repetitions	ROM ($\circ$)	Velocity ($\circ$/s)
Active shoulder abduction	10	$94.21 \pm 4.38$	$19.53 \pm 3.92$
Straight leg raise	12	$77.64 \pm 7.25$	$15.86 \pm 3.44$
Air squat	3	$118.49 \pm 1.68$	$22.18 \pm 4.97$
Bodyweight deep squat	7	$105.41 \pm 5.53$	$24.61 \pm 6.28$
Half squat (angled view)	2	$34.43 \pm 2.35$	$8.17 \pm 2.61$

ROM and angular velocity ($\circ$/s) are reported as mean ± standard deviation.

It should be noted that the number of repetitions reported in Table 8 is lower than in Table 7. This is because Table 7 reports the raw accuracy of the repetition counting algorithm and therefore includes all repetitions detected within a sequence. In contrast, Table 8 evaluates per-repetition biomechanical parameters (ROM and angular velocity), which require stable, complete movement cycles with clear joint trajectories. Repetitions that were truncated, affected by occlusion, or displayed jitter in keypoint tracking were excluded to avoid biasing the biomechanical statistics. For example, in the Bodyweight Deep Squat exercise, although 15 repetitions were available for counting, only 7 exhibited stable knee angle trajectories suitable for reliable ROM and velocity computation.

Accurate repetition detection is crucial for reliable monitoring and performance feedback in physical therapy. By consistently identifying the start and end of each movement, the system enables meaningful analysis and comparison with ideal movement models, thereby enhancing its clinical utility.

The inclusion of ROM and velocity variability provides further confidence in the system’s ability to deliver consistent biomechanical feedback across multiple repetitions and exercise types.

### Comparison with RepNet

RepNet, a neural network designed to detect repetitions in videos using artificial intelligence, is evaluated as a potential tool to identify repetitions within exercise recordings. An open-source implementation of RepNet available at GitHub^35^ is used for local testing. The primary objective is to compare RepNet’s repetition-counting accuracy against an angle-based method that utilizes joint movement data to determine repetitions as depicted in Table 9.

**Table 9:** Comparison of repetition counting accuracy between the angle-based method and RepNet across different exercise types.

Exercise type	Angle-based reps	RepNet reps	Actual reps
Active shoulder abduction with small weight (3 reps)	3	3	3
Active shoulder abduction	1	0	1
Active straight leg raise (3 reps)	3	0	3
Air squat	4	4	4
Bodyweight deep squat	4	0	4
Bodyweight half squat (angled view)	3	0	3
Bodyweight squat (chair supported)	3	3	3
Bodyweight squat (angled view)	5	4	5
Bodyweight squat (chair video 2)	2	0	2
Incorrect	1	0	1
Movement inaccurate (bending elbow and using body)	3	1	3
Movement inaccurate (bending elbow)	3	2	3
Movement OK but too fast	4	4	4
Shoulder abduction (neutral)	2	2	2
Shoulder abduction (supinated)	2	2	2
Shoulder abduction with a band	4	4	4
Shoulder abduction with a band 2	3	0	3
Straight leg raise (3 reps)	3	3	3
Straight leg raise (angled view)	3	1	3
Straight leg raise (lower raise and slightly angled view)	5	4	5
Swiss ball curl up	3	3	3
Swiss ball jack knife (3 reps)	3	3	3
Swiss ball jack knife	4	4	4

The table presents the number of repetitions detected by both methods and compares them with the actual number of repetitions performed.

The analysis of the results highlights significant limitations in RepNet’s ability to accurately count repetitions across various exercises. In multiple cases, RepNet fails to detect any repetitions, particularly for exercises such as Active Straight Leg Raise (3 reps), Bodyweight Deep Squat, Bodyweight Half Squat (angled view), and Bodyweight Squat (Chair Video 2). The angle-based method, on the other hand, successfully identifies the correct number of repetitions for these movements, aligning perfectly with the actual counts. Additionally, for exercises involving slight variations in movement, such as Movement Inaccurate (bending elbow and using body) and Straight Leg Raise (Angled View), RepNet tends to undercount repetitions, whereas the angle-based method consistently provides accurate results.

One key advantage of the angle-based approach lies in its ability to focus on specific joint movements relevant to each exercise. Unlike RepNet, which attempts to extract repetition information from the entire video without explicit knowledge of joint motion, the angle-based method leverages predefined kinematic data to track movements with precision. This allows the angle-based method to differentiate between relevant and irrelevant motions, leading to more accurate repetition detection.

Furthermore, computational efficiency is a notable concern when using RepNet. The model requires substantial processing time to analyze videos, which limits its suitability for real-time or near-real-time applications. In contrast, the angle-based approach operates more efficiently, as it directly extracts and processes joint angles without the need for complex video feature extraction and neural network inference.

Another critical limitation of RepNet is its inability to determine whether a complete repetition has been executed correctly. Since it lacks direct biomechanical awareness, it cannot assess movement quality or determine whether a repetition meets predefined clinical standards. This shortcoming is particularly relevant in rehabilitation settings, where ensuring proper form is crucial to preventing injury and optimizing recovery outcomes. These findings suggest that the angle-based method outperforms RepNet for repetition detection; its superior accuracy, computational efficiency, and ability to assess joint-specific movement patterns make it a more reliable tool for tracking exercise performance in a clinical context.

### Empirical validation

The empirical validation of the system is conducted through a series of experiments designed to assess its accuracy, effectiveness, and practical utility in a clinical setting. The evaluation process involves testing the system with a dataset comprising video recordings of various rehabilitation exercises. The results demonstrate the system’s capability to provide precise, real-time feedback, reinforcing its potential to enhance physical therapy practices. The validation outcomes confirm the system’s reliability, with key performance metrics exhibiting a high correlation with expert assessments and minimal deviations from expected performance.

A critical aspect of the validation process is the assessment of system performance in measuring key biomechanical parameters. To verify the accuracy of the automated scoring system, ROM and movement velocity are compared with expert clinician measurements, as these metrics are essential to evaluate movement quality and identify abnormalities. The analysis reveals that the automated system closely aligns with expert evaluations, demonstrating its robustness in tracking human movement.

Table 10 presents a comparative analysis of ROM measurements between the automated system and the clinician assessments. The results indicate minor deviations, with errors ranging from 1.6% to 3.2%. These findings highlight the system’s precision in capturing joint articulation and suggest that automated calculations are reliable for clinical applications.

**Table 10:** Validation of ROM and movement velocity against clinical measurements.

Joint	Automated ROM ($\circ$)	Clinician ROM ($\circ$)	Deviation (%)
Right shoulder	88.2	90.0	1.8
Left shoulder	89.5	91.0	1.6
Right hip	120.3	124.0	3.0
Left hip	119.8	123.5	3.0
Right knee	140.7	145.0	3.1
Left knee	139.9	144.5	3.2

The differences indicate minor deviations between automated calculations and expert annotations.

To complement this, Table 11 reports per-repetition statistics (mean ± SD) of ROM and angular velocity across subjects and trials. Unlike the absolute ROM values in Table 10, which represent full movement excursions, the values in Table 11 correspond to per-cycle variations derived after segmentation and temporal alignment using DTW. These reflect cycle-to-cycle variability rather than the total joint excursion, which explains the lower magnitudes observed (e.g., 15–30°). All participants performed full-range exercises as demonstrated in the reference videos; the reduced values in Table 11 therefore capture intra-trial fluctuations around the mean trajectory rather than limited motion. The values, therefore, quantify the consistency, smoothness, and repeatability of execution rather than the maximum achievable joint angles.

**Table 11:** ROM and velocity statistics from automated system across subjects and repetitions.

Joint	ROM ($\circ$)	ROM SD ($\circ$)	Velocity ($\circ$/s)	Velocity SD ($\circ$/s)
Right shoulder	15.64	13.61	7.96	11.15
Right hip	27.81	42.03	4.38	13.48
Right knee	21.02	11.64	9.37	9.36

Values are reported as mean ± standard deviation.

Both Tables 10 and 11 report joint angles measured in the sagittal plane, corresponding to flexion–extension movements at the shoulder, elbow, hip, and knee. This plane of motion was chosen because the exercises analyzed predominantly involve sagittal-plane articulation, which provides the most clinically relevant measure of range of motion for physical therapy assessment.

In addition to ROM analysis, movement velocities are examined by computing the rate of angular change over time (in degrees per second). The system employs a central difference method to estimate angular velocity while minimizing noise artifacts. The computed angular velocity profiles are subsequently compared with the movement speeds assessed by the clinician, yielding an average error margin of approximately 0.1$\circ$/s. This further supports the system’s accuracy in detecting movement velocity and its ability to maintain consistency with expert evaluations.

The successful validation of the action scoring system underscores its significant potential for real-world clinical applications. Its ability to provide accurate, real-time feedback can enhance patient adherence to prescribed rehabilitation exercises and improve therapeutic outcomes. Furthermore, future advancements will focus on refining the algorithm, integrating machine learning techniques for automated keypoint and joint selection, and broadening the system’s applicability to a wider range of exercises and movement patterns. Continuous development efforts can further improve the performance, reliability, and user experience of the system, solidifying its role as a valuable tool in clinical rehabilitation.

## Discussion

The action scoring algorithm evaluates the accuracy of a user’s exercise performance by comparing their movements with pre-stored ideal models. This process leverages two key techniques: DTW and NCC. DTW measures the similarity between two temporal sequences by aligning the user’s movement patterns with the ideal model. DTW effectively handles variations in movement speed, providing a robust comparison.

Similarly, NCC assesses the similarity between two sequences by measuring their relative displacement. It evaluates how closely the user’s movements match the ideal trajectory, ensuring a detailed analysis of the movement patterns. It calculates the angles formed by three keypoints defining the joint being exercised to count repetitions. Peaks in the angle trajectory represent the midpoint of each repetition, while signal inversion identifies the start and end points. A padding technique aligns the peaks of movements for both user and ideal data, ensuring precise repetition-by-repetition comparison.

For comparison and feedback, processed user data is evaluated against ideal data using DTW and NCC methods. Discrepancies between the performed and ideal movements are identified, enabling feedback to help users correct their posture or improve movement quality. This approach significantly enhances the evaluation of exercise execution, which is crucial for effective rehabilitation outcomes. The integration of DTW and NCC within the action scoring system has demonstrated significant accuracy and reliability in evaluating exercise performance. The DTW method effectively aligns temporal sequences, accommodating variations in movement speed, which is crucial for a fair comparison between patient and demonstration videos. The high correlation coefficients between DTW metrics and expert evaluations underscore the robustness of this approach. In addition, NCC further enhances the system’s capability by assessing the similarity between movement trajectories. The high average NCC scores indicate that the patient’s movements closely mirror ideal models, validating the effectiveness of the normalization and feature extraction processes. These results suggest that the combined use of DTW and NCC provides a comprehensive measure of movement accuracy, aligning well with clinical assessments.

The comparison with the RepNet model provides a valuable baseline for evaluating repetition counting performance. While RepNet offers an automated mechanism for detecting repetitions, our results indicate that the angle-based approach achieves higher precision and lower error margins across multiple exercise types. This suggests that angle-based repetition detection may be better suited for clinical applications where accurate movement segmentation is critical. The observed discrepancies also underscore the importance of tailoring repetition counting strategies to the nature of the movement and available sensor data.

The proposed system offers several key advantages. The system reduces the subjectivity inherent in manual evaluations by utilizing automated techniques. This leads to more consistent and repeatable assessments, crucial for tracking patient progress over time. The combination of DTW and NCC provides a multi-faceted evaluation of exercise performance. This dual approach captures various aspects of movement, such as temporal alignment and trajectory similarity, offering a comprehensive assessment. Furthermore, the system’s capability to provide immediate feedback based on quantitative scores allows for timely adjustments during therapy sessions. Patients and clinicians can quickly identify and correct deviations from prescribed exercises, enhancing the overall efficacy of rehabilitation.

Although joint angles are computed directly from pose keypoints, the angular-based approach in our system offers increased robustness to specific categories of disturbance, particularly global jitter, minor translation noise, and scaling inconsistencies. This resilience stems from the fact that joint angles are derived from relative geometric relationships between neighboring keypoints (e.g., shoulder–elbow–wrist), rather than relying on absolute positional coordinates.

In scenarios where pose estimation is affected by uniform spatial shifts (such as camera jitter or bounding box drift), angular relationships between joints remain stable because all keypoints are displaced proportionally. As a result, angular features are less sensitive to such disturbances compared to coordinate-based trajectory features, which are directly impacted by framewise positional variation. Nonetheless, we acknowledge that angular metrics remain vulnerable to more severe issues like occlusion or keypoint dropout. To mitigate this, we incorporate bounding box normalization and keypoint visibility thresholds during preprocessing. While our results demonstrate promising accuracy under typical conditions, a more formal robustness evaluation using controlled keypoint noise or occlusion scenarios is an important direction for future work.

Although the proposed framework demonstrates promising accuracy, it is important to acknowledge that the skeletal model provided by MediaPipe is inherently simplified. The system represents the human body with 33 keypoints, which do not capture the full complexity of spinal articulation or the true anatomical centers of rotation for major joints. Such abstraction can introduce minor discrepancies when compared to clinical ground truth. Nevertheless, our validation results (Tables 7, 8, 9, 10) show that the derived joint angles, ranges of motion, and movement velocities remain closely aligned with clinician measurements, indicating that the simplified skeletal structure is sufficient for rehabilitation monitoring. Moreover, the reduced keypoint set allows for computational efficiency and real-time performance, which are critical for clinical deployment. While future work may incorporate more biomechanically detailed models to refine joint center estimation and spinal dynamics, the current pose estimation framework provides an effective and clinically adequate foundation for automated movement analysis.

Despite its strengths, the proposed system has certain limitations. It may struggle with highly complex or multi-joint movements involving significant degrees of freedom. Further refinement of feature extraction and alignment techniques is necessary to accommodate such complexities. Patients with severe mobility impairments may exhibit movements that are difficult to align and compare using the current algorithms. Customization and adaptive techniques might be required to support a broader range of patient conditions. The system’s accuracy also depends on the quality of input video data; poor lighting, occlusions, or low-resolution footage can adversely affect pose estimation and subsequent analysis. Additional challenges are expected when deploying the system in real-world clinical environments. These include greater variability in patient movement patterns, irregular or incomplete motion sequences, a higher likelihood of occlusion due to assistive devices or postural variations, and unpredictable environmental factors such as camera positioning and lighting conditions. Addressing these challenges will require more robust keypoint detection models, enhanced filtering techniques, and adaptive feedback mechanisms tailored to individual users.

To address these limitations, several areas for future research are identified. Integrating more advanced pose estimation models, such as those based on deep learning, could enhance keypoint accuracy, especially in challenging conditions. Developing adaptive algorithms that adjust to individual movement patterns and patient variability would further improve robustness and clinical utility. Enhancing the system’s feedback mechanisms with machine learning techniques could also yield more personalized and actionable insights. Furthermore, large-scale clinical validation studies will be essential to confirm effectiveness across diverse populations. The addition of wearable motion sensors could offer multimodal input, enriching the accuracy and scope of movement analysis.

While our current evaluation compares the proposed method with RepNet, a widely used video-based repetition counting model, we acknowledge that this single comparison does not fully represent the broader landscape of rehabilitation technologies. Sensor-based systems, such as IMU^20^ and Kinect-based solutions^26^, have proven effective in clinical settings. Other HPE-based frameworks, such as those using HRNet^36^, OpenPose^37^, have also been applied in physiotherapy contexts. Expanding the evaluation to include these systems would provide a more nuanced understanding of the method’s comparative performance, which we recognize as a critical direction for future work.

The proposed action scoring system represents a significant advancement in the objective evaluation of physical therapy exercises. It provides a robust, scalable solution for assessing movement accuracy and adherence by combining DTW, NCC, and a lightweight pose estimation pipeline. While limitations remain, the encouraging results and clear avenues for enhancement suggest that this system can play a valuable role in clinical rehabilitation. Continued development and validation will likely yield even greater benefits in supporting effective, data-driven therapy interventions.

## Conclusion and future work

This study presents an advanced action scoring system for physical therapy exercises, integrating MediaPipe-based pose estimation with DTW and NCC to enhance movement assessment accuracy. The system effectively quantifies exercise correctness, movement alignment, and repetition counting while providing real-time feedback to support rehabilitation monitoring. Experimental results validate the system’s reliability in keypoint detection, demonstrating an average deviation of less than 10% from ground-truth data across various physical therapy exercises. Furthermore, the repetition counting mechanism, utilizing angular calculations to identify peaks and troughs in joint movements, achieved an average error of approximately 7.5%, ensuring an accurate assessment of exercise compliance and intensity. Notably, the angle-based repetition counting method outperformed the RepNet model in both accuracy and computational efficiency, making it more suitable for real-time applications. The integration of DTW and NCC further strengthened movement alignment and trajectory comparison, enabling detailed and objective performance evaluation. Beyond accuracy, the system’s ability to provide automated, real-time feedback has significant clinical implications. Assisting patients in maintaining correct movement patterns enhances exercise adherence, improves rehabilitation outcomes, and increases patient engagement. Moreover, by reducing the dependency on in-person physiotherapy sessions, the system alleviates strain on healthcare providers and facilitates remote rehabilitation monitoring, increasing accessibility to physical therapy services.

### Future work

Future research will incorporate machine learning models for adaptive keypoint selection and automated movement classification to enhance the system’s effectiveness. Expanding the capability to analyze more complex, multi-joint movements will also be a priority, ensuring broader applicability in physical therapy and sports rehabilitation. Additionally, enhancing feedback mechanisms through personalized recommendations and improving computational efficiency will make the system more practical for widespread deployment in both clinical and home-based environments.

A key future challenge will be testing robustness in real-world rehabilitation scenarios, where patient movements are often irregular, unpredictable, and subject to occlusions. Larger and more diverse clinical studies will be needed to confirm system performance under these conditions. Another direction is incorporating patient-specific anthropometric data. Since MediaPipe provides only image-based keypoints, it does not capture differences in body proportions or limb lengths. Adding simple calibration steps or clinical measurements could improve accuracy and personalization.

We also plan to validate the system against independent, high-accuracy motion capture solutions such as Optical Motion Capture (OMC). Finally, as the current approach is best suited for single-plane movements, future work will explore multi-view or depth-sensing methods to extend the system to full 3D joint kinematics while preserving its low-cost, real-time advantages.

## Data Availability

The datasets generated and/or analysed during the current study are not publicly available due to privacy concerns but are available from the corresponding author on reasonable request. The movement data were obtained from professionally filmed demonstrations and self-performed actions by the authors, with no patient data involved.
